# Fully automated detection of paramagnetic rims in multiple sclerosis lesions on 3T susceptibility-based MR imaging

**DOI:** 10.1016/j.nicl.2021.102796

**Published:** 2021-08-27

**Authors:** Carolyn Lou, Pascal Sati, Martina Absinta, Kelly Clark, Jordan D. Dworkin, Alessandra M. Valcarcel, Matthew K. Schindler, Daniel S. Reich, Elizabeth M. Sweeney, Russell T. Shinohara

**Affiliations:** a Penn Statistics in Imaging and Visualization Endeavor (PennSIVE) Center, Department of Biostatistics, Epidemiology, and Informatics, University of Pennsylvania, Philadelphia, PA, USA; b Translational Neuroradiology Section, National Institute of Neurological Disorders and Stroke (NINDS), National Institutes of Health (NIH), Bethesda, MD, USA; c Department of Neurology, Cedars-Sinai Medical Center, Los Angeles, CA, USA; d Department of Neurology, Johns Hopkins School of Medicine, Baltimore, MD, USA; e Department of Psychiatry, Columbia University Medical Center, New York, NY, USA; f New York State Psychiatric Institute, New York, NY, USA; g Department of Neurology, University of Pennsylvania, Philadelphia, PA, USA; h Department of Population Health Sciences, Weill Cornell Medicine, New York, NY, USA; i Center for Biomedical Image Computing and Analytics, Department of Radiology, University of Pennsylvania, Philadelphia, PA, USA

**Keywords:** PRL, paramagnetic rim lesion, Multiple sclerosis, Neuroimaging, Paramagnetic rim lesions

## Abstract

**Background and Purpose:**

The presence of a paramagnetic rim around a white matter lesion has recently been shown to be a hallmark of a particular pathological type of multiple sclerosis lesion. Increased prevalence of these paramagnetic rim lesions is associated with a more severe disease course in MS, but manual identification is time-consuming. We present APRL, a method to automatically detect paramagnetic rim lesions on 3T T2*-phase images.

**Methods:**

T1-weighted, T2-FLAIR, and T2*-phase MRI of the brain were collected at 3T for 20 subjects with MS. The images were then processed with automated lesion segmentation, lesion center detection, lesion labelling, and lesion-level radiomic feature extraction. A total of 951 lesions were identified, 113 (12%) of which contained a paramagnetic rim. We divided our data into a training set (16 patients, 753 lesions) and a testing set (4 patients, 198 lesions), fit a random forest classification model on the training set, and assessed our ability to classify paramagnetic rim lesions on the test set.

**Results:**

The number of paramagnetic rim lesions per subject identified via our automated lesion labelling method was highly correlated with the gold standard count per subject, r = 0.86 (95% CI [0.68, 0.94]). The classification algorithm using radiomic features classified lesions with an area under the curve of 0.82 (95% CI [0.74, 0.92]).

**Conclusion:**

This study develops a fully automated technique, APRL, for the detection of paramagnetic rim lesions using standard T1 and FLAIR sequences and a T2*phase sequence obtained on 3T MR images.

## Highlights

• Paramagnetic rim lesions are an important subtype of multiple sclerosis lesion.
• Automated methods can accelerate the assessment of paramagnetic rim lesions.
• APRL automatically identifies and accurately classifies paramagnetic rim lesions.

## 1 Introduction

Multiple sclerosis is a demyelinating and inflammatory disorder whose hallmark is lesions in the brain and spinal cord (Sahraian and Radü, 2007). These lesions can be detected *in vivo* with MRI and are often quantified as total lesion volume and lesion count, both of which can be used as measures of disease burden and to track disease progression (Popescu et al., 2013). Imaging biomarkers such as these are commonly used in the clinic and as surrogate endpoints in clinical trials (Filippi and Agosta, 2010, Sormani and Bruzzi, 2013). However, other known biological processes of MS are left uncaptured.

Chronic active lesions, a subset of MS lesions that are more prevalent in patients with more severe disease (Absinta et al., 2019, Frischer et al., 2015, Luchetti et al., 2018), have imaging and histopathology findings suggestive of ongoing tissue damage (Dal-Bianco et al., 2017, Absinta et al., 2016, Kaunzner et al., 2019, Gillen et al., 2021) and have until recently only been detectable by histopathology. These lesions have also been termed as slowly expanding, or smoldering lesions. At an estimated prevalence of as low as 4% but up to 10-15% of all MS lesions, this type of lesion is sufficiently common and deleterious to warrant considerable efforts for biomarker development (Frischer et al., 2015, Dal-Bianco et al., 2017, Absinta et al., 2016, Chawla et al., 2018). On T2*-phase MRI contrast, they are identifiable by curvilinear hypointensity along the edge of the lesion that corresponds with iron laden phagocytic cells observed on histopathological specimens (Dal-Bianco et al., 2017, Absinta et al., 2016, Bagnato et al., 2011). Here, we refer to them as paramagnetic rim lesions (PRLs).

When first observed on MRI, the rim of a PRL was only visible on scans from ultra-high-field strength (7T) magnets (Absinta et al., 2013, Bian et al., 2013, Mehta et al., 2013, Hammond et al., 2008). Recently, PRLs have been shown to be identifiable on the more common high-field strength (3T) MRI scans as well, albeit with lower inter- and intra-rater reliability (Absinta et al., 2018). This development strengthens their viability as a target on clinical MRI protocols, particularly because the sequences studied can be acquired with high spatial resolution in less than 4 minutes (Sati et al., 2014). Previous studies of PRLs have noted the geometric nature of the rim and worked to identify the rim on the quantitative susceptibility mapping contrast as well (Eskreis-Winkler et al., 2015, Stüber et al., 2016, Wisnieff et al., 2015).

Manual inspection of MS lesion for the presence of a paramagnetic rim is difficult, time consuming, and prone to inter- and intra-rater variability. We propose an automated method for identifying PRLs that would improve efficiency of study and facilitate translation of this biomarker into larger research studies and clinical practice. One way to identify PRLs is through the quantification of visual patterns that characterize these data. Radiomics is an emerging field of research that encompasses the extraction of quantitative features from biomedical images that may reflect underlying pathophysiology (Rizzo et al., 2018). Studies have shown that radiomic features are often useful predictors of known hallmarks of disease (Coroller et al., 2016, Liu et al., 2016, Bakas et al., 2017, Sweeney et al., 2021), although they have not been used extensively in the MS literature. We use radiomic features along with a random forest classification model, which can flexibly model high dimensional data, to identify PRLs. Our method is fully automated and uses a T2*-phase volume with isometric voxels and high spatial resolution that is acquired in a clinically feasible acquisition time at 3T (Sati et al., 2014).

## 2 Materials and methods

### 2.1 Study population

We studied 20 subjects with MS who were scanned under an institutional review board–approved natural history protocol at the National Institutes of Health (NIH), who were included in this study due to the presence of visible PRLs in MR scans. Subjects’ age at the time of scanning ranged from 20 to 66 years, with a mean age of 45 years (sd = 12) (Table 1). Written informed consent was obtained from all participants. Data from this study can be shared upon reasonable request and completion of a Data Transfer Agreement with the National Institutes of Health.

**Table 1:** Demographics of Study Sample.

**Demographics**	
N	20
Age (mean (SD))	45.5 (12.4)
Male (%)	8 (40)
Phenotype (%)	
Primary progressive MS	3 (15)
Relapsing-remitting MS	12 (60)
Secondary progressive MS	5 (25)
Disease Duration (mean (SD))	15.1 (9.0)
EDSS (median (range))	2.5 (1.0–7.0)
Treatments (%)	
Untreated	6 (30)
Glatiramer acetate	1 (5)
Interferon beta-1a	4 (20)
Dimethyl fumarate	6 (30)
Fingolimod	1 (5)
Natalizumab	1 (5)
Rituximab	1 (5)

### 2.2 MR Imaging acquisition:

All subjects were imaged on a Siemens Magnetom Skyra (Siemens, Erlangen, Germany) 3T scanner, using a body transmit coil and a 32-channel receive array coil, at the National Institutes of Health in Bethesda, Maryland. Imaging acquisition included the following sequences:

• a whole-brain 3D T2-weighted fluid-attenuated inversion recovery (FLAIR) sequence (repetition time, TR = 4800 ms; echo time, TE = 354 ms; inversion time, TI = 1800 ms; flip angle, FA = 120°; acquisition time, TA = 6 minutes 30 seconds; 256 axial slices; 1mm isometric voxel resolution),
• a whole-brain 3D T1-weighted magnetization-prepared rapid gradient echo (T1) sequence (TR = 7.8 ms; TE = 3 ms; FA = 18°; TA = 3 minutes 35 seconds; 256 sagittal slices; 1mm isometric voxel resolution), and
• a 3D segmented echo-planar imaging (EPI) sequence with whole-brain coverage providing T2* magnitude and phase contrasts (TR = 64 ms; TE = 35 ms; flip angle, FA = 10°; TA = 5 minutes 46 seconds; 251 sagittal slices; 0.65mm isometric voxel resolution).

Additional standard MRI sequences, including a postcontrast 3D T1-weighted MPRAGE sequence for the identification of gadolinium-enhancing lesions, were also acquired but not incorporated into the automated assessment of PRLs.

### 2.3 Manual paramagnetic rim lesion assessment:

Supratentorial non-gadolinium enhancing MS lesions were visually inspected for the presence of a paramagnetic rim on T2* magnitude and unwrapped phase images by a neurologist with 14 years of experience in neuroimaging science (Absinta et al., 2019, Absinta et al., 2013, Absinta et al., 2018). Gadolinium enhancing lesions were excluded from the analysis because the main focus of this paper was to study chronic rim lesions. In Absinta et al. (2016) (Absinta et al., 2016), PRLs were found in 22 out of 40 gadolinium enhancing lesions. Of these 22, 45% of the rims disappeared within 3 months after enhancement resolved. As previously described (Yao et al., 2012), we identify a PRL when a hypointense signal on phase images is observed surrounding the periphery of the lesion, while being either hyper- or isointense in its inner portion. PRLs were delineated on the phase with a line through the center of the lesion along its longest axis on an axial slice.

### 2.4 Image preprocessing

Phase images were unwrapped and filtered as previously described (Absinta et al., 2013). T1, FLAIR, and phase images were preprocessed using the *fslr* R package (Muschelli et al., 2015), an R wrapper for the FSL software (Jenkinson et al., 2012, Smith et al., 2004), further described below. Images were visualized with ITK-SNAP (Yushkevich et al., 2006). The T2*-magnitude contrast was not used in this method.

To preprocess our images, we first applied the N4 inhomogeneity correction algorithm to the T1, FLAIR, and phase images (Tustison et al., 2010). We then rigidly registered both the T1 and the FLAIR images to the T2*-phase image space, resampling to 0.65 mm isometric resolution and using a mutual information cost function and sinc interpolation. When deciding on registration parameters, we also considered using 9-parameter and 12-parameter registration but found that registration with those degrees of freedom resulted in some failed cases with warped images. We used multi-atlas skull stripping (MASS) to identify cerebral tissue in the images in T1 space (Doshi et al., 2013). In two cases, MASS yielded poorly skull-stripped images based on visual inspection. For those two cases, we instead used the FSL brain extraction tool for skull-stripping (Jenkinson et al., 2012). As a final step, we performed WhiteStripe intensity normalization on the otherwise preprocessed T1, FLAIR, and phase images (Shinohara et al., 2014).

### 2.5 Lesion labelling

Our lesion labelling method relies on access to maps that represent voxel-wise probabilities of being a lesion. We use the automatic lesion segmentation method MIMoSA for its ability to integrate multimodal information and to provide voxel-wise probability maps (Valcarcel et al., 2018). Manual lesion segmentation was conducted by a research assistant with 1 year of experience, who was trained by a board-certified neurologist with extensive expertise in neuroimmunology and MRI.

We trained the MIMoSA algorithm with manual segmentations as a gold standard and T1 and FLAIR images as input. We implemented a leave-one-out cross-validation approach, where data from all but one subject was used to train a MIMoSA model, and that model was subsequently applied to the remaining subject. We repeated this for every subject in our cohort.

From each k-fold model, we extracted probability maps that contained voxel-wise probabilities of being a white matter lesion. We then binarized these probability maps into lesion segmentation maps via a subject-specific estimated optimal threshold that was identified out of a user-provided range of possible thresholds and then chosen based on amount of overlap with a gold-standard lesion segmentation as measured by a Sørensen-Dice coefficient (Valcarcel et al., 2020). Because our lesion segmentation masks did not always cover the entire area of a lesion, we then dilated the masks by one voxel in each direction to increase the likelihood of detecting the paramagnetic rim signal, which occurs on the boundary of lesions. In order to then mitigate the possibility that our dilation inadvertently resulted in the added inclusion of CSF or gray matter, we used FSL FAST to segment CSF and gray matter and masked those out of the voxels newly included through dilation (Zhang et al., 2000).

**Fig. 1:**
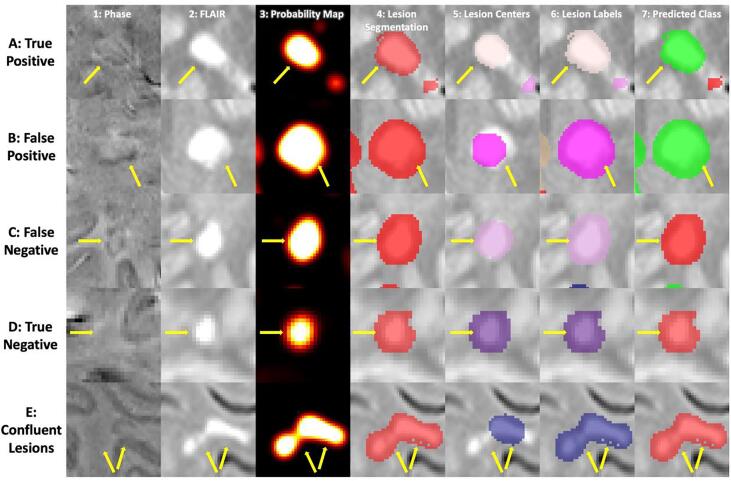
A visualization of the steps of the method for five different lesions. Each column corresponds to a different part of the method, and each row corresponds to a different lesion of interest. In columns 5 and 6, the different colors represent different lesions, where the colors are arbitrarily assigned. In the last column, lesions classified as PRLs are visualized as green, and lesions classified as not PRLs are visualized as red. Subfigure A shows a lesion that was both manually identified as a PRL and classified as a PRL, i.e. a true positive. Subfigure B shows a lesion that was manually identified as not a PRL but classified as a PRL, i.e. a false positive. Correspondingly, subfigure C shows a false negative lesion, and subfigure D shows a true negative lesion. Subfigure E shows a lesion that was automatically labelled as a single lesion but is actually a confluence of lesions.

After lesion segmentation masks were obtained, we used the lesion probability maps as input to a center detection method (Dworkin et al., 2018) to identify distinct lesions based on the texture of the lesion tissue. We then used a nearest-neighbor approach to classify the remainder of the lesion segmentation map into those identified lesions (Fig. 1). At this point, we assigned PRL status to the identified lesions based on the presence of any overlap with the manual PRL labels described previously.

Due to failures in the lesion labelling process, a subset of abnormalities automatically identified by our method might, to a manual rater, be considered clusters of confluent lesions. Because we did not have access to manual segmentations of distinct lesions, we instead relied on a combination of our lesion labelling method and connected components analysis to label lesions as confluent. Specifically, if connected components identified one cluster where our lesion labelling method identified more than one lesion, we labelled the constituent lesions as confluent.

### 2.6 Feature extraction

With the lesions identified by our automatic pipeline, we conducted a radiomic image analysis to characterize each lesion with intensity-based statistics on the phase contrast (Kolossváry et al., 2017). These include 44 features that summarize the intensities in an individual lesion in 3 general ways: by describing the average and spread of the intensities, by describing the shape of the distribution of intensities, and by describing the diversity of intensities (Kolossváry et al., 2017). For example, features like the mean, defined as $\frac{1}{n}\sum_{i=1}^{n}x_{i}$, and interquartile range, defined as $abs\left(x_{75\%}-x_{25\%}\right),$ are included in the first group, where $x_{i}$ represents intensity value at voxel i. Features like variance, defined as $\frac{1}{n}\sum_{i=1}^{n}{\left(x_{i}-mean\left(x\right)\right)}^{2}$, and skew, defined as $\frac{\frac{1}{n}\sum_{i=1}^{n}{\left(x_{i}-mean\left(x\right)\right)}^{3}}{\mathrm{sd}{\left(x\right)}^{3}}$, are included in the second group, and features like energy, defined as $\sum_{i=1}^{n}x_{i}^{2}$, uniformity, defined as $\sum_{i=1}^{n}p{\left(x_{i}\right)}^{2}$, and entropy, defined as $\sum_{i=1}^{n}-p\left(x_{i}\right)\log_{2}p\left(x_{i}\right)$, are included in the third group. A full list and detailed equations for each of the first-order radiomic features can be found in the supplemental material of Kolossváry et al. (2017).

### 2.7 Prediction model

The radiomic features were used as candidate predictors in our subsequent prediction modelling for classification of PRL lesions. Class labels for each lesion were previously assigned during the lesion labelling step. We split our dataset into a training set and test set by subject, randomly assigning lesions from 16 subjects into the training set and lesions from the remaining 4 subjects into the test set. Both sets were examined to ensure that at least 100 lesions were present in each group.

Because PRLs were of a minority class with a prevalence of approximately 12%, we used Synthetic Minority Oversampling TEchnique (SMOTE) to balance our data (Chawla et al., 2002). With SMOTE, we oversampled the PRLs by the reciprocal of the percentage of PRLs present in the dataset and we did not undersample the majority class. We then trained a random forest classifier with 10-fold cross-validation using the R package *caret* (Kuhn, 2008, Wright and Ziegler, 2017). We summarized performance results using an optimal threshold calculated based on Youden’s J statistic, which maximizes the sum of sensitivity and specificity (Youden, 1950). We also derived empirical confidence intervals for those measurements by randomly reassigning the training and test set and repeating the above process 1000 times. We assessed variable importance in the random forest as the percent increase in mean-squared error for a model with the variable over a model with a permuted version of that variable, scaled for comparability across variables.

### 2.8 Post-hoc analyses

An additional board-certified neurologist (MS) with extensive expertise in neuroimmunology and MRI, who was not involved in the generation of the manual PRL labels, examined each misclassified lesion. We rated lesions on a 5-point scale, where 1 indicated definitely not a PRL, 2 indicated probably not a PRL, 3 indicated uncertain, 4 indicated probably a PRL, and 5 indicated definitely a PRL. Some lesions were automatically labelled as one lesion but were actually a confluence of lesions (Fig. 1). We assigned manual ratings to these confluent clusters based on the presence of at least one PRL. We additionally assessed APRL’s performance only for lesions that were not part of a confluent cluster.

Because it is known that the sizes of PRLs tend to be larger than non-PRLs, we extracted lesion size for use as a potential feature in our prediction model, measured as the number of voxels in a given lesion.

## 3 Results

**Fig. 2:**
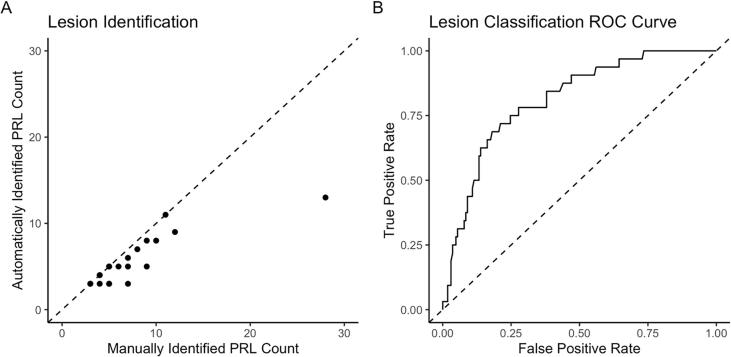
Subfigure A shows the manually identified count of PRLs against the number of PRLs estimated via our lesion identification method, r = 0.86 (0.68, 0.94). Subfigure B shows the ROC curve after classification, AUC = 0.82 (0.74, 0.92).

The final dataset included a total of 951 lesions in 20 subjects identified by our automated lesion labelling method, 113 (12%) of which we found to be PRLs by overlap with the manual annotation. The average number of lesions per subject was 47.6 (sd = 15.9), and the average number of automatically identified PRLs per subject was 5.7 (sd = 2.9). Supplementary Table 1 summarizes by subject the total number of lesions identified from our lesion labelling method, the number of PRLs identified from our lesion labelling method, and the number of PRLs identified by a manual rater. The number of identified PRLs by our method was highly correlated with the gold standard count of PRLs, r = 0.86 (95% CI [0.68, 0.94]) (Fig. 2).

We trained a random forest classification model using PRL status from the lesion labelling method as the label. In the iteration that we used to derive performance measures, there were 753 lesions in the training set, 81 of which were PRLs, and 198 lesions in the testing set, 47 of which were PRLs. Using only the undiscretized radiomic features, we were able to classify lesions with an AUC of 0.82 (95% CI [0.74, 0.92]). Using 0.502 as a probability threshold, the optimal threshold as determined by Youden’s J, 135 lesions were accurately classified as not PRL, 31 lesions were false positives, 8 were false negatives, and 24 were classified correctly as PRL (Table 2). A breakdown of the classification results for the test set lesions by subject is provided in Table 2, in which we see that the distribution of classification results are not very different between patients.

**Table 2:** Summary of Classification Performance Measures.

**Contingency Table (Excluding Confluent Lesions)**				
**Prediction**	**Reference**			

Rim Negative		Rim Positive	

Rim Negative	135 (47)		8 (0)	
Rim Positive	31 (19)		24 (6)	
**Testing Set Lesion Classification Count by Subject (Excluding Confluent Lesions)**				

Subject	True Negative	False Negative	False Positive	True Positive

1	65 (24)	4 (0)	10 (4)	4 (1)
5	13 (4)	1 (0)	8 (2)	7 (0)
8	25 (7)	0 (0)	4 (3)	5 (0)
16	32 (12)	3 (0)	9 (10)	8 (5)
**Performance Measures**	**With Confluent Lesions (95% CI)**		**Without Confluent Lesions**	

AUC	0.82 (0.74, 0.92)		0.88	
Accuracy	0.8 (0.59, 0.91)		0.74	
Positive Predictive Value	0.44 (0.17, 0.55)		0.24	
Negative Predictive Value	0.94 (0.93, 1)		1	
False Positive Rate	0.19 (0.07, 0.46)		0.29	
False Negative Rate	0.25 (0, 0.37)		0	
Sensitivity	0.75 (0.63, 1)		1	
Specificity	0.81 (0.54, 0.93)		0.71	

The table summarizes the performance measures we observed for the classification of PRLs, where counts in parentheses are counts excluding confluent lesions. 95% confidence intervals are provided for performance measures where available.

We also examined the results of the method for lesions that were not part of a confluent cluster. A total of 72 lesions in the test set were not confluent, and were able to be classified with an AUC of 0.88. Using 0.086 as the probability threshold, the optimal threshold for this subset of lesions as determined by Youden’s J statistic, 47 lesions were accurately classified as not PRL, 19 were false positive, 0 were false negative, and 6 were accurately classified as PRL (Table 2). Additional performance measures are provided in Table 2. Because we examined confluent lesions as part of a post-hoc analysis, we did not derive confidence intervals for these performance measures.

A visualization of lesions that were true positive, false positive, false negative, and true negative respectively is provided in Fig. 1. From subfigure B, where we see the method illustrated for a lesion that was falsely identified as a PRL, we can see that hypointensities can manifest around a lesion even when they cannot be rated as a rim. Conversely, from subfigure C, which shows a lesion that was falsely identified as not a PRL, we see that despite the presence of hypointensities that are visible to the eye, certain PRLs may not display a signal strong enough to be captured by radiomic features.

**Fig. 3:**
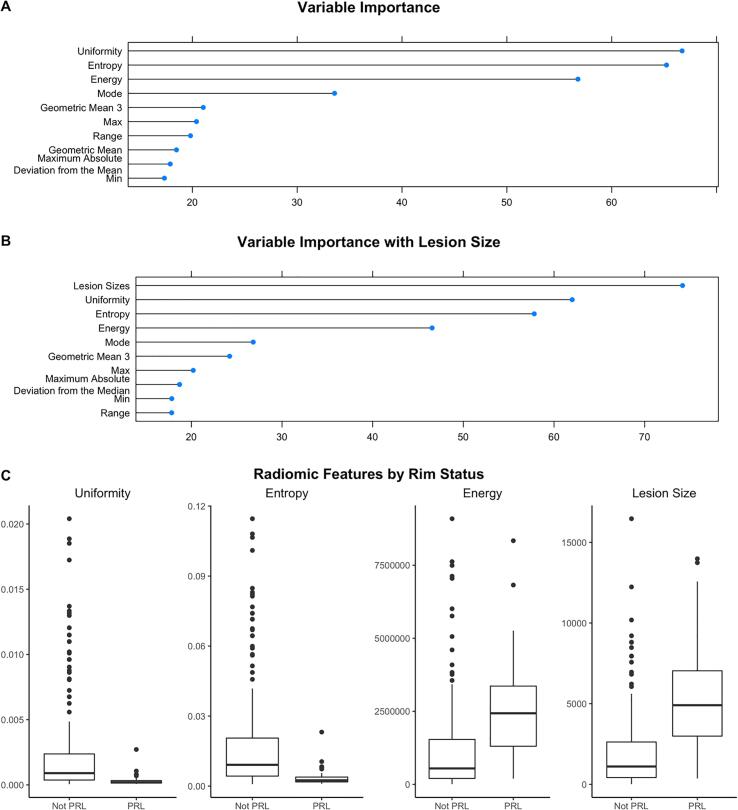
The variables identified as the most important by APRL for determining the presence of PRLs were uniformity, entropy, and energy. Here, we measure variable importance as the percent increase in mean squared error for the model with the variable over the model with a permuted version of that variable, scaled for comparability across variables. Boxplots of uniformity, entropy, energy, and lesion size on the lesions from the test set show that PRLs and non-PRLs seem to differ on those measures, supporting the theory that they are important for distinguishing the two kinds of lesions.

The random forest identified uniformity, entropy, and energy as the most important radiomic features for classifying lesions, which are all features that aim to describe the diversity of the data points (Fig. 3). Other radiomic features that were important were mode, kurtosis, and skew. Entropy and uniformity were both higher in lesions that were not PRLs, and energy was higher in PRLs. In a model including lesion size as an additional predictor, the random forest identified lesion size as the most important feature, with PRLs expressing larger sizes than lesions that were not PRLs, predicting PRL status with an AUC of 0.81. A model using textural features classified lesions with an AUC of 0.72.

A second expert manually rated the 39 lesions that were misclassified by the model. The rater deemed that 1 lesion included too much artifact to assess PRL status, and 25 lesions were confluent. Of the lesions not part of a confluent cluster, 9 were false positive and 5 were false negative. Of those 9 false positive lesions, 4 were rated as definitely a PRL, 2 were rated as uncertain, 2 were rated as probably not a PRL, and 1 was rated as definitely not a PRL. For the 5 false negative lesions that were not confluent, 1 was rated as definitely a PRL, 2 were rated as probably a PRL, 1 was rated as uncertain, and 1 was rated as probably not a PRL.

As for confluent clusters, 22 were false positives and 3 were false negatives. These were rated according to the presence of at least one PRL in each confluent cluster. Of the 22 false positive lesions, 11 were rated as definitely a PRL, 5 were rated as probably a PRL, 1 was rated as uncertain, 3 were rated as probably not a PRL, and 2 were rated as definitely not a PRL. All 3 of the false negative lesions were rated as definitely a PRL. We note that the confluence defined here was a judgement made by the manual rater. This differs from but complements the confluence definition employed for the primary test set analysis, which was the definition based on the automated analysis used to derive the performance measures reported in Table 2.

## 4 Discussion

Preliminary studies have shown that the existence of a paramagnetic rim around an MS lesion is an important biomarker with potential clinical implications: indicative of chronic inflammation, associated with heightened disability, and resistant to current disease-modifying treatments (Absinta et al., 2019). However, paramagnetic rims are time-consuming to identify manually, even by highly trained experts (Absinta et al., 2018). In this paper, we developed APRL, a fully automatic method for detecting paramagnetic rim lesions on a 3T MRI using a submillimeter isometric, clinically feasible, segmented-EPI sequence (Absinta et al., 2018, Sati et al., 2014). Automation of PRL identification that relies on objective assessment would aid larger scaled studies assessing this promising imaging biomarker in MS. Contemporaneoulsy developed deep learning methods have also recently been published (Barquero et al., 2020).

APRL relies on radiomics for automated PRL identification and classification. Radiomic features have not previously been used to classify PRLs. The radiomic features that were the most important in this context aimed to measure the variability of intensity within a lesion (entropy and uniformity) or quantify the magnitudes of the intensities themselves (energy).

Energy measures the magnitude of intensities within a lesion. On the phase image used in this study, PRLs manifested with higher energy because hypointensities represented more extreme negative values instead of values closer to 0, with more extreme hypointensities resulting in more extreme energy values.

Both entropy and uniformity are measures based on the probability of observing a particular intensity within a lesion. Because we did not bin the voxel intensities, the number of distinct intensities observed was large, so the probability of observing a particular intensity was fairly low. This was reflected in the observed range of uniformity in this study. Uniformity is a direct measure of homogeneity of the intensities within a lesion. We expected uniformity to be lower for PRLs due to the presence of both intensities representing normal appearing tissue and hypointensities from the paramagnetic rim. Lesions that were not PRLs did not appear with any distinct signature on a phase image, leading to a higher uniformity. In addition, the impact of the size of a region of interest on radiomic features in MS lesions has not been well studied and warrants further investigation.

Entropy takes the probability of observing a particular intensity within a lesion and transforms it to reflect the amount of observed variation. Because of the aforementioned lack of binning, here, entropy more accurately reflected lesion size in that given our more homogenous set of probabilities, a smaller probability of observing a given intensity resulted in a smaller measure of entropy. Larger lesions yielded a smaller probability of observing a given intensity. In this dataset, PRLs tended to have smaller values of entropy, possibly reflecting a larger size, which has been noted in previous studies of PRLs as well (Dal-Bianco et al., 2017). When we included lesion size in our classification model, we found that lesion size was an important predictor of PRL status in addition to uniformity, entropy, and energy, suggesting that these four measures provide potentially similar but nevertheless complementary forms of information for classifying PRLs. but nevertheless complementary forms of information for classifying PRLs.

Many of the lesions that the model misclassified were confluent lesions that were labelled as a single lesion. According to our automated assessment of confluence, the percentage of confluent lesions among correctly classified lesions was 33%, while the percentage of confluent lesions among incorrectly classified lesions was 49%, suggesting that confluence negatively influences the model’s ability to classify PRLs. According to our expert rater’s visual assessment of confluence, nearly 65% of misclassified lesions were confluent. Of these, 88% were false positives, potentially reflective of heterogeneity in intensity that is more present for confluent lesions but also in lesions with a rim signal. Confluent lesions also tend to be larger, similar to PRLs, which may have also contributed to the misclassification.

We provide an example of one of these confluent lesions in Fig. 1, Subfigure E. In this lesion, although one of the encompassed lesions contains a clear rim signal, the larger of the two does not. Because the majority of the voxels included in the confluent lesion belong to the encompassed one without a rim signal, the first-order radiomic features extracted from this confluent lesion reflected that signal.

We dilated our lesion segmentation map to increase the likelihood that a rim signal would be included in a lesion label. In order to mitigate the impact of inclusion of ventricular and cortical phase-hypointensities, we masked out cerebrospinal spinal fluid and gray matter from the dilations, but this dilation could have nevertheless resulted in the inclusion of non-lesional tissue that may have affected the calculation of radiomic features.

These issues could be addressed by taking a more nuanced approach to modelling the probability of having a rim. Here, we treated the identification of PRLs as a binary classification problem, invoking a random forest to predict if a given lesion was a PRL. However, the identification of PRLs can be difficult because of the myriad of factors that drive the clarity and strength of a rim signature, some of which are technical and some of which reflect biological processes. As noted in Fig. 1, while some lesions exhibit a rim unequivocally, other lesions exhibit a more equivocal signature. This renders the task of identifying PRL lesions difficult, both for manual raters and automated classifiers. In fact, previous research has shown that intra- and interrater reliability for paramagnetic rim evaluation are substantial but not perfect, with a Cohen κ of 0.77 and 0.71 respectively (Absinta et al., 2018). A future approach could treat the presence of a rim as a continuous measure instead of a binary classification, where middling levels of this theoretical measure could represent both uncertainty about a lesion’s classification and different stages of PRL progression. This would likely more accurately reflect underlying biological processes as well, as the amount of iron-containing phagocytes at the edge of a lesion can vary across lesions (Dal-Bianco et al., 2017).

### 4.1 Limitations

A major limitation to current assessments of paramagnetic rims is that no international consensus exists on criteria for determining this imaging signature. This limitation may hinder the application of the proposed methodology to new studies in which differing definitions of paramagnetic rims may be desired based on local practices. While signal-to-noise ratio is higher on a 7T MR image, allowing for higher inter- and intra-rater reliability, they remain low across contrast types on 3T (Absinta et al., 2018). However, APRL relies on techniques that perform well on 3T images, so extensions to 7T would require additional validation.

This study may be improved by the collection of additional data containing delineations of rim signal locations. Increasing the sample size may allow for a more accurate reflection of the imaging signature associated with PRLs within the feature space, and a more specific delineation of the rim signal may improve APRL’s ability to differentiate between hypointensity due to the presence of a rim and hypointensity due to noise or features like the central vein sign. In the current study, we did not explicitly assess for the presence of a central vein sign in each of the automatically identified lesions. Because the central vein sign also presents as hypointensity within a lesion on T2*-phase, a central vein sign might impact the calculation of first-order radiomic features. Textural features, which quantify the spatial relationship between voxel intensities (Haralick et al., 1973), characterized PRLs less accurately than first-order features. Future studies may explore more direct methods for quantifying the central vein to disentangle the rim signal and the central vein sign. In addition, all the patients for this analysis had at least one PRL. Given recent histology work (Gillen et al., 2021), we do not suspect that patient without PRL lesions would have different radiomic signatures in their non-PRL lesions from patients with PRL lesions, but further work is warranted to investigate this.

Additionally, in the current study, we did not explicitly consider gadolinium-enhancement in our automated identification of PRLs. Gadolinium-enhancing lesions were specifically left out of the manual assessment in an effort to specifically study chronic rim lesions, whose presence has previously been shown to be associated with poor prognostic factors (Absinta et al., 2019). Paramagnetic rims in gadolinium-enhancing lesions fade within 3 months in a high percentage of cases (Absinta et al., 2016) and may exhibit features different from chronic rim lesions on imaging due to edema and tissue architecture, though this was not explicitly studied in this analysis.

## 5 Conclusion

This study introduces a fully automated method, APRL, for the identification and classification of paramagnetic rim lesions relying solely on 3T MR images, which are commonly available in a clinical setting. Automation of this process is important for the continued development of the scientific community’s knowledge around these lesions and their implications for disease burden.

## Funding

Dr. Pascal Sati, Dr. Martina Absinta, and Dr. Daniel S. Reich are supported by the Intramural Research Program of the National Institute of Neurological Disorders and Stroke, National Institutes of Health, Bethesda, Maryland, USA. Dr. Martina Absinta is supported by the Conrad N. Hilton Foundation (grant#17313). Dr. Schindler is supported by the National Center for Advancing Translational Sciences of the National Institutes of Health under award number KL2TR001879. Ms. Lou and Dr. Shinohara are supported by awards R01NS112274 and R01NS060910 from the National Institute of Neurological Disorders and Stroke, and R01MH112847 from the National Institute of Mental Health. The content is solely the responsibility of the authors and does not necessarily represent the official views of the National Institutes of Health.

## CRediT authorship contribution statement

**Carolyn Lou:** Methodology, Software, Formal analysis, Investigation, Writing – original draft, Visualization. **Pascal Sati:** Conceptualization, Resources, Writing - review & editing. **Martina Absinta:** Resources, Resources, Data curation, Writing - review & editing. **Kelly Clark:** Data curation. **Jordan D. Dworkin:** Methodology, Software, Writing - review & editing. **Alessandra M. Valcarcel:** Methodology, Software, Writing - review & editing. **Matthew K. Schindler:** Validation, Writing - review & editing. **Daniel S. Reich:** Conceptualization, Writing - review & editing. **Elizabeth M. Sweeney:** Methodology, Writing – original draft, Supervision. **Russell T. Shinohara:** Conceptualization, Methodology, Writing – original draft, Supervision.

## Declaration of Competing Interest

A.M.V. is currently an employee of Genentech but did not receive funding or consulting fees as it pertains to this work. D.S.R. receives unrelated research funding from Vertex Pharmaceuticals. R.T.S. receives consulting fees from Octave Bioscience, and receives compensation for reviewing scientific articles from the American Medical Association and for reviewing grants for the Emerson Collective, National Institutes of Health, and the Department of Defense.
